# Molecular characteristics of *Staphylococcus aureus* associated prosthetic joint infections after hip fractures treated with hemiarthroplasty: a retrospective genome-wide association study

**DOI:** 10.1038/s41598-020-73736-3

**Published:** 2020-10-06

**Authors:** J. Christopher Noone, Marc Stegger, Berit Lilje, Knut Stavem, Karin Helmersen, Inge Skråmm, Hege Vangstein Aamot

**Affiliations:** 1 Department of Microbiology and Infection Control, Akershus University Hospital, 1478 Lørenskog, Norway; 2 Faculty of Medicine, University of Oslo, Oslo, Norway; 3 Department of Bacteria, Parasites and Fungi, Statens Serum Institut, Copenhagen, Denmark; 4 Department of Pulmonary Medicine, Akershus University Hospital, Lørenskog, Norway; 5 HØKH, Department of Health Services Research, Akershus University Hospital, Lørenskog, Norway; 6 Department of Clinical Molecular Biology (EpiGen), Akershus University Hospital and University of Oslo, Lørenskog, Norway; 7 Department of Orthopedic Surgery, Akershus University Hospital, Lørenskog, Norway

**Keywords:** Microbiology, Medical research

## Abstract

A retrospective study of *Staphylococcus aureus* isolates from orthopaedic patients treated between 2000 and 2017 at Akershus University Hospital, Norway was performed using a genome-wide association approach. The aim was to characterize and investigate molecular characteristics unique to *S. aureus* isolates from HHA associated prosthetic joint infections and potentially explain the HHA patients’ elevated 1-year mortality compared to a non-HHA group. The comparison group consisted of patients with non-HHA lower-extremity implant-related *S. aureus* infections. *S. aureus* isolates from diagnostic patient samples were whole-genome sequenced. Univariate and multivariate analyses were performed to detect group-associated genetic signatures. A total of 62 HHA patients and 73 non-HHA patients were included. Median age (81 years vs. 74 years; *p* < 0.001) and 1-year mortality (44% vs. 15%, *p* < 0.001) were higher in the HHA group. A total of 20 clonal clusters (CCs) were identified; 75% of the isolates consisted of CC45, CC30, CC5, CC15, and CC1. Analyses of core and accessory genome content, including virulence, resistance genes, and k-mer analysis revealed few group-associated variants, none of which could explain the elevated 1-year mortality in HHA patients. Our findings support the premise that all *S. aureus* can cause invasive infections given the opportunity.

## Introduction

Femoral neck fractures among the elderly are frequent, with an estimated overall incidence rate in Norway of 189 per 100,000 person years^1^. The common treatment of displaced intracapsular femoral neck fractures is hip hemiarthroplasty (HHA)^2^. Patients undergoing HHA are, as a group, older and have a higher comorbidity burden than total hip hemiarthroplasty (THA) patients^3,4^, a risk factor commonly referred to as frailty^5^. This makes them more vulnerable to detrimental health outcomes such as surgical complications. A 2016 meta-analysis found the rate of prosthetic joint infections (PJIs) among HHA patients to be between 1.7 and 7.3%, making it a salient problem for both the patient as well as the healthcare system, given the frequency of the procedure. Increased risk of infection, and consequently mortality, has been shown in hip fracture patients with prolonged preoperative wait^6^. Onset of a HHA-associated PJI increases the already high risk of 1-year mortality from 25 to 50% in this patient group^7^.

However, a significantly lower incidence of 1-year mortality has been reported in similar femoral neck fracture patients treated with THA instead of HHA^8^. Furthermore, an unexplained discord between HHA-patient infection rates and their NNIS (The Center for Disease Control’s National Nosocomial Surveillance system) risk scores has been reported^9^. NNIS risk assessment is used to predict a patients’ risk of developing a surgical site infection (SSI)^10^. The NNIS indexes correspond well to the SSI rate among non-HHA implant patients^11^.

Several studies have reported *S. aureus* as the most frequent pathogen causing HHA infections^12,13^. HHA infections have been reported to be distinct from THA, both clinically and microbiologically^7^, thus prompting questions of risk-associated microbial variation among HHA-associated PJIs. The aim was to characterize and investigate molecular characteristics unique to *S. aureus* isolates from HHA-associated prosthetic joint infections and potentially explain the HHA patients’ elevated 1-year mortality compared to a non-HHA group.

## Methods

### Study design and setting

This retrospective genome-wide association study was conducted at Akershus University Hospital (Ahus). Ahus is the largest acute-care hospital in Norway serving about 500,000 people (~ 10% of the Norwegian population) and performs approximately 4,000 orthopaedic implant surgeries annually.

### Patients and comparison groups

All patients > 18 years old suffering from *S. aureus* implant-related infections after hip fractures treated with hemiarthroplasty at Ahus from 2000 to 2017 were eligible for inclusion.

The comparison group was made up of all patients with *S. aureus* implant-related infections following one of four types of surgery in lower extremities at Ahus within the same period: total hip arthroplasty (THA), total knee prosthesis (TKA), implantation of headless compression screws (HCS) for trochanteric fractures, and osteosynthesis of ankle implants.

Both HHA and non-HHA patients with pathological fractures and polymicrobial infections were excluded.

Patient data such as age at the time of surgery, gender, date of death, sample date, ASA (American Society of Anesthesiologists) classification^14^, surgery duration time, and wound contamination class were collected from the patients’ electronic medical records. Being a retrospective study, patient data not entered at the time of treatment were not retrievable and listed simply as missing data.

NNIS risk indexes were calculated from ASA classification, surgery duration, and wound class^10^. ASA classification is the anaesthesiologist’s pre-surgical assessment of a patient’s fitness at the time of surgery. The 75th percentile of an operation’s duration is used to determine if the duration of a procedure had been prolonged to the point of increased risk of infection. Except for hip arthroplasty, no national data for the 75th percentile of orthopaedic surgery duration exists. Therefore, the 75th percentile of surgery duration was calculated locally from the surgery times of each of the operation types presented here (Supplementary Table S1). To better facilitate multivariable analysis, some ASA and NNIS classifications with few instances were merged: NNIS indexes 2 and 3; ASA classifications 1 and 2, and classifications 3 and 4.

### Culturing and DNA extraction

Upon cultivation and identification as a part of routine diagnostics, all *S. aureus* isolates were stored at − 80 °C. After overnight culturing at 35 °C, isolates were pre-treated with 10 µl lysostaphin dissolved in 190 µl PBS, and incubated at 37 °C for 30 min. DNA was then extracted using QIAsymphony DNA Mini Kit and the QIAsymphony SP according to the manufacturer’s protocol (Qiagen, Hilden, Germany).

### Whole-genome sequencing

DNA library prep was accomplished using the Nextera XT DNA Library Preparation Kit and Nextera XT Index Kit (Illumina, San Diego, USA) using standard protocol (Illumina document #15031942v03), but working volumes up to and including library amplification were halved; library clean up volumes were reduced to 20 µl PCR product and 10 µl of Agencourt Ampure XP beads (Beckman Coulter Inc., Atlanta, Georgia). Quantification of libraries was carried out using KK4835 KAPA Library Quantification Kit qPCR (Roche, Rotkreuz, Switzerland). The 300-bp paired-end, whole-genome sequencing was performed using Illumina’s MiSeq platform and MiSeq v3 Reagent Kit. The generated sequencing data were subjected to quality control using bifrost (https://github.com/ssi-dk/bifrost) which ensures adequate sequencing depth of all isolates. Check for contamination was performed using Kraken v1.0^15^ prior to assembly with SPAdes v3.11.1^16^ using default settings. All contigs were used and the assembly metrics can be found in the supplemental data (Supplementary Table S2).

### Genome wide analyses

FASTQ files from the MiSeq runs were assessed using NASP v1.0 (Northern Arizona SNP pipeline)^17^. Duplicate regions were filtered from analysis using NUCmer v1.0^18^ and mapped to the *S. aureus* CA347 reference chromosome (GenBank accession ID NC_021554) via Burrows-Wheeler Aligner^19^. Core single nucleotide polymorphisms (SNPs) were called using GATK v 4.0.11.0^20^. Minimum depth was set at 10 and SNP consensus to minimum 90% to ensure high-quality SNPs for downstream analyses.

Sequence types were extrapolated from assembled genome data and clonality determined using a multilocus sequence type (MLST) allelic profile query (https://saureus.mlst.net/sql/allelicprofile_choice.asp).

**Figure 1:**
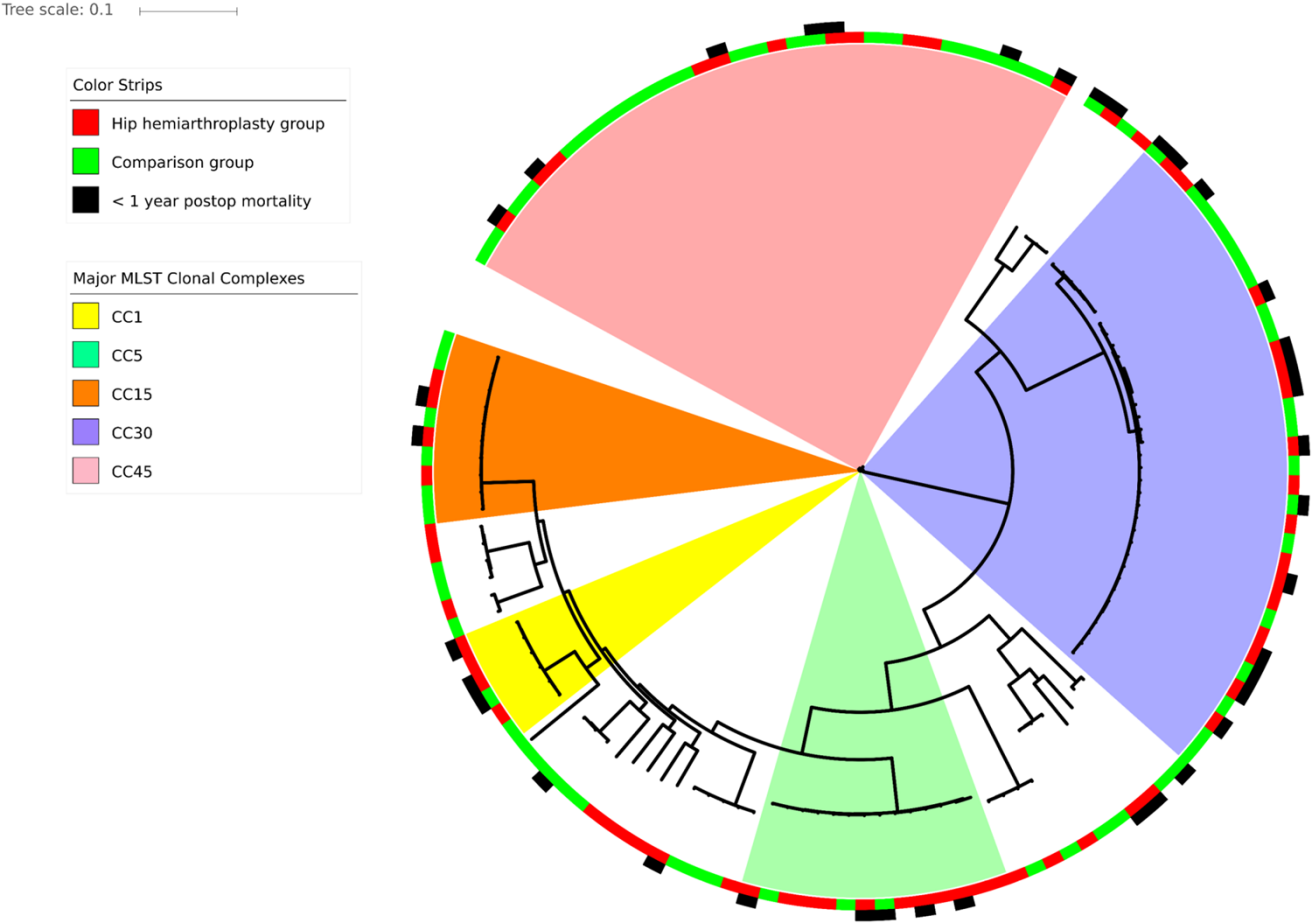
A centre rooted SNP based phylogenetic tree of all *S. aureus* isolates from hip hemiarthroplasty (n = 62) and comparison group (n = 73) isolates collected at Akershus University Hospital between 2000 and 2017. The tree is based on core genome SNPs visualized in iTOL v4.2.3. (https://itol.embl.de/) (https://itol.embl.de/). The coloured sectors indicate the five dominant clonal complex types. Colour strips along the circumference indicate group affiliation (red and green) and, where relevant, 1-year all cause postoperative mortality (black).

The NASP SNP matrix was the input for a phylogenetic tree constructed using a maximum likelihood approximation in FastTree v2.1.5^21^. The phylogeny was rooted at mid-point and visualized using iTOL v4.2.3. (https://itol.embl.de/) (Fig. 1).

Multivariate SNP analysis was carried out using Discriminant Analysis of Principle Components (DAPC) v2.1.1. in the R package *adegenet*^22^. Open reading frames were identified with Prokka v1.2^23^ with default settings and then analyzed by comparing the annotated genomes using Roary v3.6.2^24^, with a minimum percentage identity for BLASTP of 90 to investigate gene presence/absence across the data set. To facilitate downstream analysis, positions identical to the reference were set to ‘0′, positions displaying polymorphisms were set to ‘1’; positions displaying two or three variations (3.2%) were also set to ‘1’.

The existence of group-associated SNPs in the accessory genome was investigated using univariate analysis (Fisher’s exact test) and multivariate analysis via the open source R packages *treeWAS*^25^ and *pcadapt*^26^.

### K-mer analysis

K-mer analysis^27^ using a 30-bp k-mer length was performed to search for group-associated variants. Significantly associated k-mers were aligned with the reference genome and annotated in CLCbio’s Genomics Workbench (www.clcbio.com) to determine the loci.

### Resistance and virulence factors

An unbiased approach to finding risk associated patterns of genetic traits in general was performed was performed using DAPC and k-mer analysis. The isolates were further profiled for antimicrobial resistance and virulence markers using ResFinder v3.2 (https://cge.cbs.dtu.dk/services/ResFinder/) and VirulenceFinder v2.0 (https://cge.cbs.dtu.dk/services/VirulenceFinder/).

### Statistical analysis

Groups were compared using the Student’s t-test for continuous variables, Mann–Whitney U-test for ordinal variables, or Fisher’s exact test for categorical variables.

Logistic regression was performed to compare the 1-year mortality between the HHA patients and the non-HHA patients adjusted for gender, age, NNIS indexes, and ASA classifications, which were considered as possible confounding variables of the association between group affiliation and 1-year mortality.

Statistical analysis on patient demographics and clinical data was performed using SPSS v23 (IBM SPSS, Armonk, NY, USA). Significance was set to *p* = 0.05 and the *p*-values of all bioinformatics results were corrected for multiple testing using the false discovery rate (FDR) method^28^ or Bonferroni correction^29^.

### Ethics approval

This work was approved by the Data Protection Official at Akershus University Hospital (PVO 16-140) who concluded that this study was exempt from the additional ethical approval of the Regional Committee for Medical Ethics. Consent has, thereby, been waived by the Data Protection officials. The bacterial isolates were cultured from patient samples taken during conventional diagnostics, and all work was done in strict accordance to the ethical guidelines in place at the time of study approval.

## Results

### Patient demographics and clinical data

In total, 62 HHA patients and 73 non-HHA patients were included. The non-HHA group included 34 THA, 6 TKA, 22 HCS, and 11 ankle osteosyntheses. Patient demographics are presented in Table 1.

**Table 1:** Patient demographics and risk factors of the study’s hip hemiarthroplasty (HHA) and comparison group (non-HHA) patients collected at Akershus University Hospital 2000–2017.

	HHA (n = 62)	Non-HHA (n = 73)	*p*-values
Gender, male/female	22/40	34/39	0.194
Total hip arthroplasty		15/19	
Total knee arthroplasty		2/4	
Headless compression screws		9/13	
Ankle		8/3	
Age at surgery, median [range]	81 [61–98]	74 [24–104]	< 0.001
**Surgery type, n (%)**			
Acute surgery	62 (100)	39 (65)	< 0.001
Elective surgery	0	22 (35)	
**ASA**^**a**^** classification, n (%)**			
1	0	5 (7)	0.001
2	21 (34)	40 (55)	
3	37 (60)	25 (34)	
4	3 (5)	1 (1)	
**NNIS**^**b**^** risk index, n (%)**			
0	19 (31)	30(42)	0.373
1	31 (50)	30 (42)	
2	11 (18)	9 (13)	
3	0	2 (3)	

^a^ASA, American Society of Anesthesiologists classification.

^b^NNIS, The Center for Disease Control’s National Nosocomial Surveillance system.

The median preoperative wait for the HHA group was 27 h (5–168 h). The infections of the HHA group were categorized as 90% early infections (< 3 months) and 10% sub-acute infections (3–24 months). The non-HHA comparison group’s infections were categorized as 93% early, 6% sub-acute, and 1% late onset (> 24 months). The categorical distributions were not significantly different between the patient groups.

The 1-year mortality in the HHA group, 27 of 62 (44%), was higher than that of the comparison group, 11 of 73 (15%) (*p* < 0.001) (Table 1). 1-year mortality was limited to two operation types in the comparison group: THA (n = 2, 6%), and HCS (n = 9, 40%). After adjusting for gender, age, NNIS indexes, and ASA classifications, HHA cases were still at higher odds of dying within one year of their surgery than non-HHA patients (odds ratio 3.61; 95% confidence interval 1.45–8.95: *p* = 0.006).

Data missing from the patients’ electronic records included data specifying whether 12 (16%) of the THA surgeries were elective or acute; 3 ASA classifications, 1 (1%) HHA patient and 2 (3%) non-HHA patients; and finally, components needed for NNIS calculations for 1 (1%) HHA patient and 2 (3%) non-HHA patients.

### Clonality

In total, 20 MLST-based clonal complexes (CCs) were identified. The most frequent were CC45, CC30, CC5, CC15, and CC1 (Table 2). No CC was associated with either group.

**Table 2:** Distribution of the clonal complexes and resistance genes in patients with *S. aureus* infections across the hip hemiarthroplasty (HHA) group (n = 62) and the non-HHA comparison group (n = 73).

Clonal complex, n (%)	HHA	Non-HHA
45	11 (18)	24 (33)
30	16 (26)	18 (25)
5	10 (16)	3 (4)
15	4 (6)	6 (8)
1	5 (8)	4 (5)
Others^a^	16 (26)	18 (25)
**Resistance gene, n (%)**		
*aac(6′)-aph(2′')*	0	1 (1)
*blaZ*	45 (72)	51 (70)
*cat(pC221)*	1 (2)	1 (1)
*dfrG*	3 (5)	1 (1)
*erm(A)*	1(2)	0
*erm(C)*	2 (3)	0
*erm(T)*	2 (3)	1 (1)
*mecA*	1 (2)	0
*mph(C)*	1 (2)	0
*msr(A)*	1 (2)	0
*spc*	1 (2)	0
*tet(K)*	4 (6)	2 (3)
*tet(M)*	0	0
Total resistance genes	62	57

^a^Others: CCs 6, 7, 8, 9, 12, 20, 22, 25, 49, 50, 59, 72, 97, 121, and 398.

### Gene presence or absence

A total of 4,421 putative genes were detected across the population using Roary. Of these, 1,717 of these were present in 99% or more of the isolates and perceived to be the core genome, the remaining 2,704 putative genes inferred as the accessory genome. Univariate testing (Fisher’s exact test) and multivariate analysis (DAPC) of the accessory genome revealed no genes significantly associated with either of the patient groups.

### SNPs

**Figure 2:**
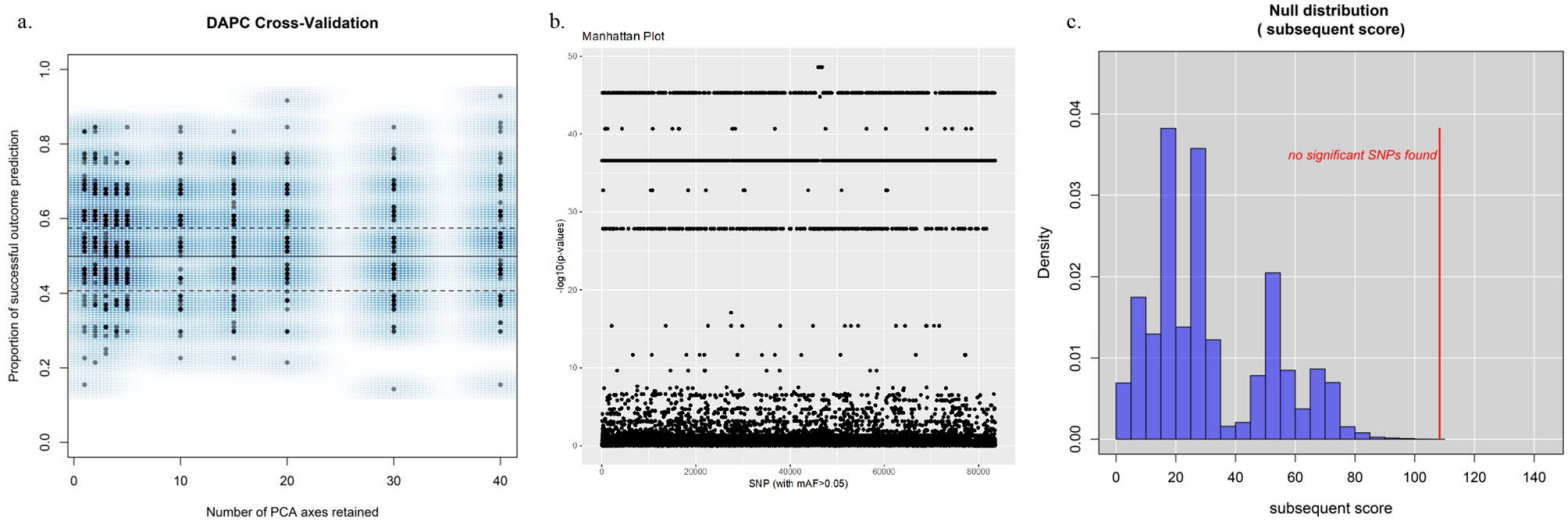
Diagrams from GWAS-based results of single nucleotide polymorphisms variation between HHA (n = 62) and non-HHA comparison (n = 73) groups. (**a**) DAPC cross validation plot: the X-axis indicates the number of principal components retained in the analyses of each respective column, and the Y-axis the ability of each principal component analysis to predict the samples’ group affiliation; each point represents a repetition of the analysis. (**b**) DAPC Manhattan plot of all detected SNPs across both the HHA and non-HHA groups; numbers along the X-axis indicate SNP number; the negative logarithm of the association *p*-value for each SNP is displayed on the Y-axis. Each point on the Manhattan plot represents a SNP. Outliers represent SNPs from which case isolates are significantly over- or under-represented relative to controls. (**c**) Null distribution subsequent test output from *treeWas*: the subsequent score measures the proportion of tree branches where group affiliation matches an associated genotype. The red line indicates significance threshold, above which real patient group-correlated risk variants are indicated, thus no group of SNPs retained its significant group association after *treeWAS* controlled for the confounding effects of lineage. It has been rendered using R software v3.6.1, (https://www.r-project.org/), packages *adegenet* and *treeWAS.*

Analysis revealed 83,661 SNPs from the core genome of the 135 isolates. Neither univariate nor multivariate analysis yielded SNPs significantly associated with either group. Isolates, regardless of their patient group status, clustered according to clonal complex (Fig. 1). No number of principle components performed better than approximately random as illustrated in the DAPC cross validation plot (Fig. 2a). Predicative power ranged from 0.46 to 0.56.

DAPC analysis produced seven significant groups of outlier SNPs (Fig. 2b), which were, apparently, the result of the slight overrepresentation of certain lineages in either the HHA group or the non-HHA group. The signal disappeared after analysis with *treeWAS* (Fig. 2c), which distinguishes between true genotype-associated variation and the confounding effects of lineage-associated variation^25^.

To address the possibility of circulating clones, an investigation into the SNP-based relatedness of the isolates was performed with a threshold of 50 SNPs that identified a group of three identical isolates. Four pairs of isolates with fewer than 50 SNPs were also detected with two, 13, 29, and 44 SNPs differentiating them. In total, 11 isolates (8%) were found to be closely related, five from the HHA group and six from the non-HHA group.

### K-mer analysis

K-mer analysis yielded 50 significant k-mers that aligned to four genomic sequences, all of which were associated with the non-HHA comparison group. The first of the four mapped to a 33 nucleotide region partially overlapping the tRNA gene for asparagine (*p* = 0.001); the second to a 49 nucleotide region which partially overlapped the gene encoding the tRNA for isoleucine (*p* = 0.031); the third aligned to a 42 nucleotide region overlapping the gene encoding the tRNA for alanine (*p* = 0.031); finally, the fourth aligned to a 30 nucleotide region located at the 3ʹ-end of the gene coding for the 5S rRNA subunit (*p* = 0.006). The aligned k-mer sequences are detailed in the supplemental data (Supplementary Table S3).

### Resistance and virulence factors

Despite unbiased approaches to find risk-associated patterns of genetic traits yielding nothing significant, ResFinder and VirulenceFinder results profiles of each group are also included. ResFinder analysis detected 13 different resistance genes; their distribution is detailed in Table 2. The most frequently detected was *blaZ* occurring in 72% of HHA and 70% non-HHA group isolates.

Analysis of VirulenceFinder results yielded three genes that were overrepresented in the HHA group: *sed* (*p* = 0.006), *sej* (*p* = 0.012), and *ser* (*p* = 0.006). The isolates positive for these genes (9 HHA group and 1 non-HHA), contained all three or none, except in the case of one HHA isolate which was positive for only *sed* and *ser*. These genes were no longer significantly associated after correction for multiple testing. The isolates positive for these genes clustered together on the phylogenetic tree with CC5 (eight isolates) and CC8 (two isolates). Detailed results of the virulence factors present are included in the supplementary data (Supplementary Table S4).

## Discussion

No distinguishing pattern of clonality, virulence, or resistance were found in the genomes of *S. aureus* isolates from the HHA group. Because no risk-associated variants were discovered, efforts to explain the elevated 1-year mortality in HHA PJI patients and generate hypotheses for follow-up studies were abandoned despite that HHA PJI patients in our study showed significantly higher 1-year mortality even after controlling for the possible confounding effects of age and patient health. The 1-year mortality of 44% was in accordance with other studies^12,13^. The work of others have shown an association between prolonged preoperative wait and increased risk infection and mortality^6^. We found no such association in the HHA group. Median preoperative wait for the HHA group was 26.5 h, which resembled more closely the median preoperative wait of the non-infected group (26 h) in the aforementioned study. The preoperative waits of the HHA and non-HHA groups were deemed incomparable because 35% of the non-HHA patients were undergoing elective procedures with a predetermined preoperative wait.

The most frequently encountered CCs in both the HHA and comparison groups were CC45, CC30, CC5, CC15, and CC1, which are commonly encountered lineages in Norway both in other invasive infections as well as nasal carriers^30,31^.

There was, as expected, a large degree of SNP diversity among the isolates. Five groups of isolates had fewer than 50 SNPs between them. Apart from a group of three, these groups came in pairs, and operation dates of the patients in question did not coincide; thus, indicating that these infections were not due to any major outbreak but rather local acquisition or even carriage due to their general presence as prevalent human colonizing lineages in Norway.

Gene presence/absence indicated no group-specific patterns of resistance or virulence genes. The most frequently detected resistance gene was *blaZ*, which encodes a beta-lactamase, an enzyme that hydrolyses penicillin^32^. The distribution of the *blaZ* was similar in the HHA (72%) and non-HHA (70%) cohorts, and the frequencies were similar to those found in the invasive infections of previous work^31^. The virulence genes (*sed*, *sej*, and *ser*) that were slightly overrepresented in the HHA group clustered along the lines of phylogeny, indicating that their association is most likely the result of the effects of lineage rather than any association to phenotype. The three genes code for staphylococcal enterotoxins responsible for toxinoses such as food poisoning and toxic shock syndrome^33,34^ No signal of these genes was detected from the unbiased gene presence/absence analysis due to the extensive correction for multiple testing. After correction for multiple testing, these genes were no longer significant in this targeted approach.

K-mer analysis found a significant over-representation of 50 k-mers in the non-HHA comparison group which partially aligned to four genes: those encoding asparagine tRNA, isoleucine tRNA, alanine tRNA, and the 5S rRNA subunit. These k-mers were all associated with the isolates from the comparison group. Bacterial RNA, including tRNA and rRNA, is a frequent target for antibiotic therapies, thus inhibiting protein synthesis. Some antimicrobials target tRNA directly, and some target molecules that interact with tRNA^35–38^s. Despite this, there is no record of such antimicrobials being administered to these patients.

The 5S sub-unit is a virtually ubiquitous part of the ribosome in all but the mitochondrial ribosomes of some fungi, higher animals, and most protists; it is thought to function as a stabilizer of the ribosome structure, and thus enhance protein synthesis^39^. The k-mers in question align to the 3′-end of five *S. aureus* 5S rRNA references in the rRNA 5S database (https://combio.pl/rrna/). Its single variation was reflected in only one of the rRNA 5S references (B02698), manifesting itself as an insertion that enlarges the loop proximal to the 3′-end of the subunit by one nucleotide. It is unclear if this variation has any impact on ribosomal function.

The strength of our study lies in the extensive genome-wide bioinformatic analyses performed on a unique collection of HHA PJI *S. aureus* isolates from cases with a relatively low incidence, collected over a span of 18 years. To our knowledge, this is the first microbial GWAS to have been performed on *S. aureus* isolates from HHA patients.

The study has certain limitations. Although the samples represent all eligible patients at Ahus from 2000 to 2017, the sample size remains small due to the aforementioned low incidence and can have resulted in suboptimal statistical power. The non-HHA comparison group was somewhat heterogeneous. However, these patients were arguably the best comparison group of patients available, having suffered *S. aureus*-associated implant infections in their lower extremities. This single centre study was also carried out in a geographical area with a low prevalence of antimicrobial resistance, limiting its generalization to areas with a greater prevalence of antimicrobial resistance.

General patient health is an integral component of the ASA assessment, which is somewhat crude. The higher median ASA classification of the HHA group, despite being controlled for with logistic regression analysis, might still indicate a trend towards generally poorer physical fitness of the members of that group, making them less robust than the comparison group, and consequently less able to tackle complications associated with acute traumatic injury and PJIs, leading to higher mortality in the HHA group.

In conclusion, clonality, virulence and resistance patterns, gene presence, and variations were virtually indistinguishable between *S. aureus* isolates from patients with HHA PJIs and non-HHA implant infections. Those differences found between the groups of isolates cannot be argued responsible for the higher incidence of 1-year mortality found in the HHA patient group. Our findings support the premise that all *S. aureus* can cause invasive infections given the opportunity.

## Supplementary information

Supplementary Tables.

## Data Availability

The whole-genome sequence data generated in this study have been submitted to the European Nucleotide Archive under Project ID Number PRJEB35740.
