# Supplementary material for: Molecular characteristics of Staphylococcus aureus associated prosthetic joint infections after hip fractures treated with hemiarthroplasty: a retrospective genome-wide association study
Source: Sci Rep. 2020 Oct 6;10:16553. doi: 10.1038/s41598-020-73736-3 (PMC7538562; doi:10.1038/s41598-020-73736-3)
Supplement: Supplementary file 1 — Supplementary Tables. [file 41598_2020_73736_MOESM1_ESM.docx]

***Supplemental data***

Molecular characteristics of *Staphylococcus aureus* associated prosthetic joint infections after hip fractures treated with hemiarthroplasty: a retrospective genome-wide association study

J. Christopher Noone^1,2^, Marc Stegger^3^, Berit Lilje^3^, Knut Stavem^2,4,5^, Karin Helmersen^1^, Inge Skråmm^6^, Hege Vangstein Aamot^1,7^

^1^Department of Microbiology and Infection Control, Akershus University Hospital, Lørenskog, Norway

^2^Faculty of Medicine, University of Oslo, Oslo, Norway

^3^Department of Bacteria, Parasites and Fungi, Statens Serum Institut, Copenhagen, Denmark

^4^Department of Pulmonary Medicine, Akershus University Hospital, Lørenskog, Norway

^5^HØKH, Department of Health Services Research, Akershus University Hospital, Lørenskog, Norway

^6^Department of Orthopedic Surgery, Akershus University Hospital, Lørenskog, Norway

^7^Department of Clinical Molecular Biology (EpiGen), Akershus University Hospital and University of Oslo, Lørenskog, Norway

| **Operation type** | **Median time** | **Range** | **75th percentile** |
| --- | --- | --- | --- |
| **Total hip arthroplasty (34)** | 100 | 65-301 | 113 |
| **Total knee arthroplasty (6)** | 84 | 66-141 | 87 |
| **Headless compression screws(22)** | 70 | 23-241 | 77 |
| **Ankle implant (11)** | 76 | 41-122 | 84 |
| **Hip hemiarthroplasty (62)** | 75 | 25-118 | 86 |

**Supplementary** **Table S1:** Operation types, number, and duration times (minutes) for all included patients.

| **Sample ID** | **Average coverage** | **Number of contigs** | **Number of reads** | **Genome size** |
| --- | --- | --- | --- | --- |
| **L1** | 28 | 270 | 422592 | 2735926 |
| **L2** | 29 | 218 | 441804 | 2655543 |
| **L3** | 25 | 437 | 359684 | 2660198 |
| **L4** | 24 | 469 | 381274 | 2682567 |
| **L5** | 43 | 89 | 880838 | 2740266 |
| **L7** | 26 | 233 | 363806 | 2630180 |
| **L8** | 39 | 80 | 807946 | 2711677 |
| **L9** | 25 | 307 | 374574 | 2704121 |
| **L10** | 28 | 267 | 399400 | 2674219 |
| **L11** | 102 | 27 | 1649948 | 2770730 |
| **L12** | 110 | 22 | 1828932 | 2716680 |
| **L13** | 41 | 80 | 880366 | 2770921 |
| **L14** | 30 | 245 | 447880 | 2665413 |
| **L15** | 24 | 442 | 351360 | 2735902 |
| **L16** | 23 | 369 | 316598 | 2635855 |
| **L17** | 38 | 116 | 798396 | 2807985 |
| **L18** | 36 | 114 | 749556 | 2708620 |
| **L19** | 24 | 447 | 372750 | 2748506 |
| **L20** | 128 | 63 | 2146854 | 2841271 |
| **L21** | 23 | 379 | 369276 | 2777687 |
| **L22** | 22 | 271 | 322150 | 2632675 |
| **L23** | 38 | 61 | 580718 | 2706449 |
| **L24** | 26 | 272 | 358168 | 2708751 |
| **L25** | 28 | 329 | 426912 | 2643529 |
| **L26** | 29 | 195 | 443008 | 2730144 |
| **L27** | 21 | 415 | 317102 | 2665427 |
| **L28** | 37 | 122 | 750464 | 2716085 |
| **L29** | 46 | 118 | 982920 | 2749423 |
| **L30** | 30 | 245 | 477228 | 2719009 |
| **L31** | 59 | 29 | 1235116 | 2733164 |
| **L32** | 23 | 275 | 313570 | 2722548 |
| **L33** | 116 | 44 | 1926206 | 2750442 |
| **L34** | 114 | 31 | 1798548 | 2742492 |
| **L35** | 104 | 24 | 1669312 | 2705397 |
| **L36** | 100 | 34 | 1632702 | 2768359 |
| **L37** | 106 | 54 | 1693672 | 2850081 |
| **L38** | 120 | 42 | 1924406 | 2734318 |
| **L39** | 113 | 25 | 1807218 | 2765323 |
| **L40** | 110 | 40 | 1751730 | 2803007 |
| **L41** | 101 | 61 | 1645296 | 2793484 |
| **L42** | 102 | 24 | 1583552 | 2714685 |
| **L43** | 108 | 44 | 1728028 | 2803164 |
| **L44** | 97 | 47 | 1510370 | 2682712 |
| **L45** | 98 | 30 | 1572928 | 2784320 |
| **L46** | 103 | 47 | 1680474 | 2784916 |
| **L47** | 118 | 28 | 1863650 | 2708691 |
| **L48** | 94 | 38 | 1455618 | 2735476 |
| **L49** | 125 | 38 | 1973474 | 2772088 |
| **L50** | 179 | 23 | 2851002 | 2735639 |
| **L51** | 108 | 25 | 1672768 | 2671303 |
| **L52** | 98 | 33 | 1580700 | 2728686 |
| **L53** | 104 | 28 | 1632630 | 2747593 |
| **L54** | 117 | 28 | 1846200 | 2747432 |
| **L55** | 121 | 27 | 1888976 | 2723818 |
| **L56** | 129 | 28 | 2016812 | 2769247 |
| **L57** | 130 | 21 | 2060000 | 2786054 |
| **L58** | 83 | 61 | 1279848 | 2716107 |
| **L59** | 100 | 36 | 1686834 | 2827918 |
| **L60** | 102 | 24 | 1625088 | 2711981 |
| **L6** | 40 | 64 | 810514 | 2667963 |
| **L61** | 36 | 100 | 739212 | 2749226 |
| **L62** | 36 | 88 | 767968 | 2814280 |
| **KTR1** | 130 | 64 | 1393602 | 2756271 |
| **KTR2** | 140 | 38 | 1563736 | 2742427 |
| **KTR3** | 153 | 57 | 1720360 | 2834507 |
| **KTR4** | 136 | 39 | 1477934 | 2673296 |
| **KTR5** | 161 | 54 | 1818138 | 2777230 |
| **KTR6** | 163 | 64 | 1912720 | 2813190 |
| **KTR7** | 154 | 61 | 1929834 | 2849127 |
| **KTR8** | 121 | 23 | 1329234 | 2718486 |
| **KTR9** | 175 | 33 | 1875810 | 2723895 |
| **KTR10** | 195 | 19 | 2299920 | 2717833 |
| **KTR11** | 154 | 44 | 1898992 | 2795985 |
| **KTR12** | 237 | 76 | 2869782 | 2894095 |
| **KTR13** | 142 | 25 | 1956360 | 2725654 |
| **KTR14** | 149 | 51 | 1845502 | 2715813 |
| **KTR15** | 146 | 32 | 1698256 | 2672719 |
| **KTR16** | 156 | 40 | 1982628 | 2735353 |
| **KTR17** | 170 | 53 | 1916468 | 2785331 |
| **KTR18** | 156 | 28 | 1657210 | 2706879 |
| **KTR19** | 145 | 25 | 1666822 | 2673968 |
| **KTR20** | 125 | 101 | 1499208 | 2866579 |
| **KTR21** | 156 | 36 | 1878374 | 2727240 |
| **KTR22** | 155 | 43 | 1881374 | 2801855 |
| **KTR23** | 143 | 38 | 1689958 | 2792199 |
| **KTR25** | 185 | 32 | 2220952 | 2685657 |
| **KTR26** | 168 | 25 | 1933726 | 2702503 |
| **KTR27** | 153 | 56 | 1775578 | 2741088 |
| **KTR28** | 138 | 62 | 1691218 | 2794436 |
| **KTR29** | 117 | 27 | 1441662 | 2770617 |
| **KTR30** | 150 | 45 | 1991450 | 2751155 |
| **KTR31** | 62 | 114 | 735290 | 2816978 |
| **KTR32** | 142 | 57 | 1827892 | 2837690 |
| **KTR33** | 162 | 42 | 1754154 | 2675801 |
| **KTR34** | 158 | 44 | 1798238 | 2736172 |
| **KTR35** | 208 | 60 | 2170280 | 2721456 |
| **KTR36** | 160 | 24 | 1815648 | 2711977 |
| **KTR37** | 129 | 62 | 1406798 | 2717335 |
| **KTR38** | 172 | 125 | 1892922 | 2763796 |
| **KTR39** | 181 | 25 | 2158056 | 2709543 |
| **KTR40** | 196 | 75 | 2117190 | 2728335 |
| **KTR41** | 173 | 27 | 2033010 | 2736641 |
| **KTR42** | 189 | 87 | 2143426 | 2729971 |
| **KTR43** | 163 | 33 | 1813390 | 2693102 |
| **KTR44** | 125 | 59 | 1431120 | 2767709 |
| **KTR45** | 255 | 57 | 3108960 | 2780411 |
| **KTR46** | 137 | 44 | 1616060 | 2723874 |
| **KTR47** | 179 | 29 | 2190646 | 2747925 |
| **KTR48** | 124 | 107 | 1549712 | 2744192 |
| **KTR49** | 123 | 84 | 1411070 | 2711671 |
| **KTR50** | 152 | 72 | 1875982 | 2756895 |
| **KTR51** | 149 | 63 | 1719632 | 2754371 |
| **KTR52** | 157 | 44 | 1867914 | 2690657 |
| **KTR53** | 198 | 45 | 2139702 | 2631732 |
| **KTR54** | 183 | 33 | 2182534 | 2759074 |
| **KTR55** | 196 | 40 | 2491138 | 2748002 |
| **KTR56** | 148 | 56 | 1802016 | 2749887 |
| **KTR57** | 170 | 138 | 1870372 | 2677961 |
| **KTR58** | 183 | 54 | 2376338 | 2708957 |
| **KTR60** | 187 | 21 | 2211398 | 2690657 |
| **KTR61** | 184 | 27 | 2406970 | 2714296 |
| **KTR62** | 197 | 39 | 2086100 | 2688750 |
| **KTR63** | 188 | 52 | 2068568 | 2684727 |
| **KTR64** | 163 | 43 | 1782132 | 2718110 |
| **KTR65** | 114 | 122 | 1259576 | 2736113 |
| **KTR66** | 100 | 134 | 1127114 | 2756979 |
| **KTR67** | 164 | 63 | 1831290 | 2715684 |
| **KTR69** | 170 | 26 | 1880798 | 2715752 |
| **KTR70** | 154 | 144 | 1643908 | 2819690 |
| **KTR72** | 136 | 135 | 1363976 | 2642270 |
| **KTR73** | 183 | 115 | 2023402 | 2831904 |
| **KTR74B** | 33 | 405 | 348194 | 2598391 |
| **KTR75** | 32 | 305 | 418068 | 2695057 |
| **KTR76B** | 31 | 506 | 335144 | 2590271 |
| **KTR77** | 38 | 458 | 405818 | 2693877 |

**Supplementary** **Table S2:** NGS assembly metrics for all *S. aureus* isolates included in this study.

| **Sequence** | **k-mers** | **Combined**  **length** | | **Locus** | | ***p*-value*** |
| --- | --- | --- | --- | --- | --- | --- |
| TAATATTAATCCACAGTAGCTCAGTGGTAGAGC | 4 | 33 | | *Asp tRNA* | | 0.001 |
| TGGTTCGAGTCCACTTAGGCCCACCATTATTTGTACATTGAAAACTAGA | 28 | 49 | | *Ile tRNA* | | 0.031 |
| ATCCCGCTAGTCTCCACCATTATTTGTACATTGAAAACTAGA | 17 | 42 | | *Ala tRNA* | | 0.031 |
| GTTCCGCTAGAGTAGAACGTTGCCAGGCAT | 1 | 30 | | *5S rRNA* | | 0.006 |
| *FDR corrected |  | |  |  |  | |

**Supplementary** **Table S3:** Details of K-mer analysis of all significant k-mer alignments across all *S. aureus* isolates, including aligned sequences, number of k-mers used in each alignment, alignment length, annotation, and FDR corrected *p*-value. These k-mers were associated to the non-HHA group.

| **Gene** | **HHA** | **non-HHA** | **Total** | ***p*-value** | ***p*-value corrected** |
| --- | --- | --- | --- | --- | --- |
| *sep* | 5 | 6 | 11 | 1 | 1 |
| *aur* | 62 | 73 | 135 | 1 | 1 |
| *edinB* | 2 | 4 | 6 | 0.687 | 1 |
| *hlgA* | 61 | 73 | 134 | 0.459 | 1 |
| *hlgB* | 62 | 72 | 134 | 1 | 1 |
| *hlgC* | 61 | 73 | 134 | 0.459 | 1 |
| *lukD* | 29 | 25 | 54 | 0.161 | 1 |
| *lukE* | 29 | 25 | 54 | 0.161 | 1 |
| *lukF-PV* | 1 | 1 | 2 | 1 | 1 |
| *sak* | 50 | 59 | 109 | 1 | 1 |
| *scn* | 54 | 65 | 119 | 0.793 | 1 |
| *sea* | 13 | 8 | 21 | 0.153 | 1 |
| *seb* | 2 | 7 | 9 | 0.178 | 1 |
| *sec* | 2 | 2 | 4 | 1 | 1 |
| *sec3* | 4 | 9 | 13 | 0.381 | 1 |
| ***sed*** | 9 | 1 | 10 | **0.006** | 0.192 |
| *seg* | 41 | 51 | 92 | 0.712 | 1 |
| *seh* | 5 | 6 | 11 | 1 | 1 |
| *sei* | 39 | 47 | 86 | 0.86 | 1 |
| ***sej*** | 8 | 1 | 9 | **0.012** | 0.384 |
| *sek* | 6 | 2 | 8 | 0.142 | 1 |
| *sel* | 6 | 6 | 12 | 0.772 | 1 |
| *sem* | 41 | 52 | 93 | 0.578 | 1 |
| *sen* | 37 | 50 | 87 | 0.367 | 1 |
| *seo* | 43 | 53 | 96 | 0.707 | 1 |
| *seq* | 6 | 2 | 8 | 0.142 | 1 |
| ***ser*** | 9 | 1 | 10 | **0.006** | 0.192 |
| *seu* | 37 | 50 | 87 | 0.367 | 1 |
| *splA* | 27 | 26 | 53 | 0.38 | 1 |
| *splB* | 29 | 22 | 51 | 0.052 | 1 |
| *splE* | 32 | 35 | 67 | 0.731 | 1 |
| *tst* | 11 | 10 | 21 | 0.635 | 1 |
| **Total** | 823 | 917 | 1740 |  |  |

**Supplementary** **Table S4:** All isolates’ virulence factors detected across the *S. aureus* isolates from both the HHA and non-HHA groups as associated to the HHA group and the *p*-values, raw and corrected for multiple testing.
